# A novel inflammatory marker for extensive ulcerative colitis; Endocan

**DOI:** 10.1186/s12876-023-02720-8

**Published:** 2023-04-11

**Authors:** Bulent Albayrak, Engin Sebin

**Affiliations:** 1 Department of Gastroenterology, Ataturk University, Erzurum, Turkey; 2 Biochemistry Department, Erzurum Regional Training and Research Hospital, Erzurum, Turkey

**Keywords:** Ulcerative colitis, Inflammatory indicator, Endocan

## Abstract

**Background & aims:**

Ulcerative colitis (UC) is an inflammatory bowel disease characterized by mucosal inflammation. Endocan, a proteoglycan secreted by endothelial cells in response to inflammatory cytokines, has been reported to be overexpressed in inflammatory conditions. In this study, we aimed to evaluate the utility of endocan level in determining the extent and severity of disease in patients with ulcerative colitis and to determine whether it can be a candidate marker for noninvasive evaluation and monitoring since there is not enough data in the literature.

**Materials and methods:**

Sixty-five people were included in the study, including thirty-five with ulcerative colitis and thirty in the control group. Patients with first diagnosed ulcerative colitis clinically, endoscopically, and histopathologically, without any treatment, and with normal liver and kidney tests were included in the study. Endoscopic scoring of all patients was performed according to the Mayo endoscopic scoring (MES) system. Blood samples for CRP (C-reactive protein) and endocan were taken from the patients simultaneously.

**Results:**

There was a significant statistical difference between all patients with ulcerative colitis and the control group in both endocan level and CRP level (*p* < 0.001). There was a statistically significant difference between endocan levels and CRP levels between the left-distal group and pancolitis (diffuse colitis) patients, but there was no statistical difference between age and MES.

**Conclusion:**

Serum endocan level can be useful in determining the extent of ulcerative colitis and planning treatment.

## Introduction

Ulcerative colitis (UC) is an inflammatory bowel disease characterized by mucosal inflammation that starts from the rectum (distal) and extends proximally, involving the entire colon (pancolitis). The Ulcerative Colitis Endoscopic Severity Index (UCEIS) is the only approved scoring system to determine the severity of the disease endoscopically, however, the Mayo score (MES) is widely used in clinical practice due to its ease of application [1].

The pathogenesis of ulcerative colitis has not been clarified yet, but it is thought to result from the irregular expression of molecules involved in proinflammatory and anti-inflammatory processes [2, 3]. Endocan, a proteoglycan secreted by endothelial cells in response to inflammatory cytokines, has been reported to be overexpressed in inflammatory conditions. As in arthritic joint tissues, high endocan levels have been demonstrated, especially in areas characterized by severe inflammation [4]. With a better understanding of the biological mechanisms that drive UC, it may be possible to find the right drug for the right person at the right time, and it may also be possible to meet the patient’s broader goals, such as the impact on quality of life [5].

In this study, we aimed to evaluate the usability of endocan level in determining the extent and severity of disease in patients with ulcerative colitis and to determine whether it can be a candidate marker for noninvasive evaluation and monitoring since there is no similar study in the literature.

## Material and method

Thirty-five patients with newly diagnosed ulcerative colitis clinically, endoscopically, and histopathologically, without any treatment, and with normal liver and kidney tests were included in the study. All patients were classified as moderate severity according to the Truelove and Witts criteria. The patients were divided into 2 groups. According to the Montreal classification, the group from the distal to the splenic flexure was classified as the left-distal group, and the group starting from the rectum and extending beyond the splenic flexure proximally was classified as pancolitis [6]. Endoscopic evaluation was performed by only one gastroenterologist. Endoscopic scoring of all patients was performed according to the mayo endoscopic scoring (MES) system [1, 7]. Thirty healthy individuals who did not have any complaints or systemic diseases and did not use any anti-inflammatory drugs were included in the control group. Patients with severe concomitant medical conditions (i.e., cardiac, renal, or hepatic failure) or any coexisting malignancy as well as pregnant women were excluded from the cohort. Blood samples for CRP (c-reactive protein) and endocan were taken from the patients simultaneously.

Samples were stored at -80 °C and all were analyzed at once to reveal the endocan level. Analyses were performed by the endocan/ESM-1 ELISA kit ChemWell Fusion 4800 automatic ELISA analyzer. The clinical research ethics committee approval of Erzurum/Turkey Regional Training and Research Hospital, date and decision number 2018/18–177, was obtained for the study.

### Statistical analysis

SPSS 20.0 (SPSS Inc., Chicago, IL, USA) program was used in the analysis of the data. In the statistical analysis of the study, the mean, standard deviation, frequency, and percentage values were defined. The general characteristics and demographic characteristics of the groups were determined by Frequency (Descriptive analysis: frequency analysis for a single variable) analysis. In pairwise comparisons; the Independent Sample t-Test was used to compare the mean of two independent groups. The Chi-square test was used to determine the relationship between categorical variables. Receiver Operating Characteristic (ROC) Analysis and Youden Index were used to determine the sensitivity and specificity of serum Endocan levels and to predict the cut-off value in the detection of diffuse colitis. Spearman Correlation was used for correlation analysis. A *p*-value of ≤ 0.05 was considered statistically significant in the entire study.

## Results

Sixty-five people were included in the study, including 35 with ulcerative colitis and 30 in the control group. The mean age of ulcerative colitis patients was 33.48, and the mean age of the control group was 32.89. There was a significant statistical difference between all ulcerative colitis patients and the control group in both endocan level and CRP levels (*p* < 0.001) (Table 1).

**Table 1:** Endocan and CRP levels of ulcerative colitis (UC) and control group

		Both Patients (*n* = 65)		
		UC (*n* = 35)	Control (*n* = 30)	*P*
Endocan	Mean	165.83 ± 137.33	59.03 ± 27.88	< 0.001*
CRP	Mean	26.74 ± 11.531	3.67 ± 2.218	< 0.001*

*CRP* C- reactive protein

*P < 0.05 is considered significant for statistical analyses.

There was a statistically significant difference between endocan levels and CRP levels between the left-distal group and pancolitis (diffuse colitis) patients (*p* < 0.001), but there was no statistical difference between age and MES (Table 2).

**Table 2:** Demographic, MES, Endocan, and CRP levels of UC patients

		Patient Groups (*n* = 35)		
		Left-Distal colitis (*n* = 16)	Pancolitis (*n* = 19)	*P*
Age	Mean	32.31 ± 5.770	34.47 ± 6.372	0.304
Endocan	Mean	46.4169 ± 17.15137	266.3916 ± 109.69373	< 0.001*
CRP	Mean	16.63 ± 4.319	35.26 ± 8.245	< 0.001*
MES	Mean	2.19 ± 0.403	2.37 ± 0.496	0.251

*MES* mayo endoscopic score

*P < 0.05 is considered significant for statistical analyses.

**Fig. 1:**
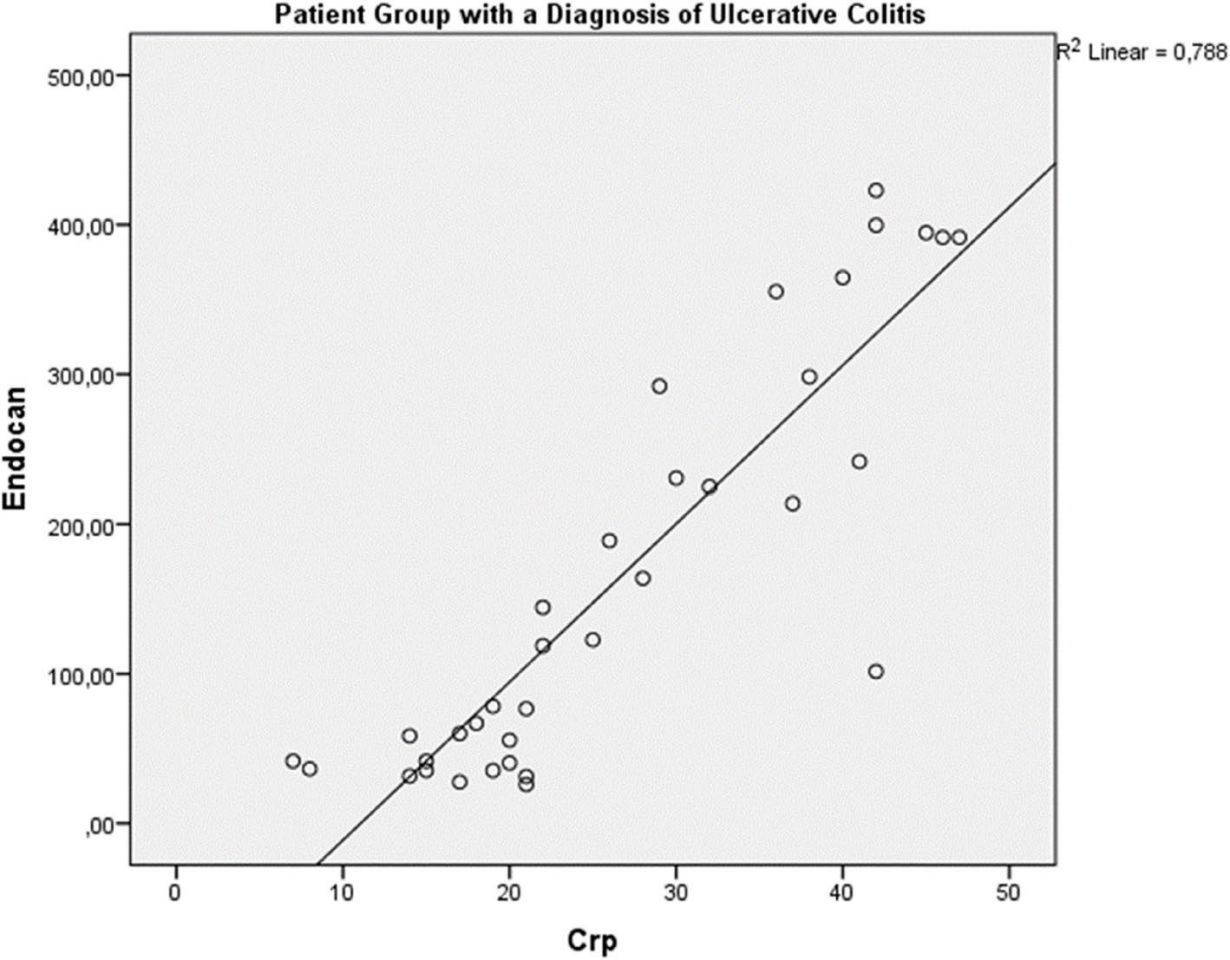
Endocan and CRP correlation analysis between all ulcerative colitis patients

**Fig. 2:**
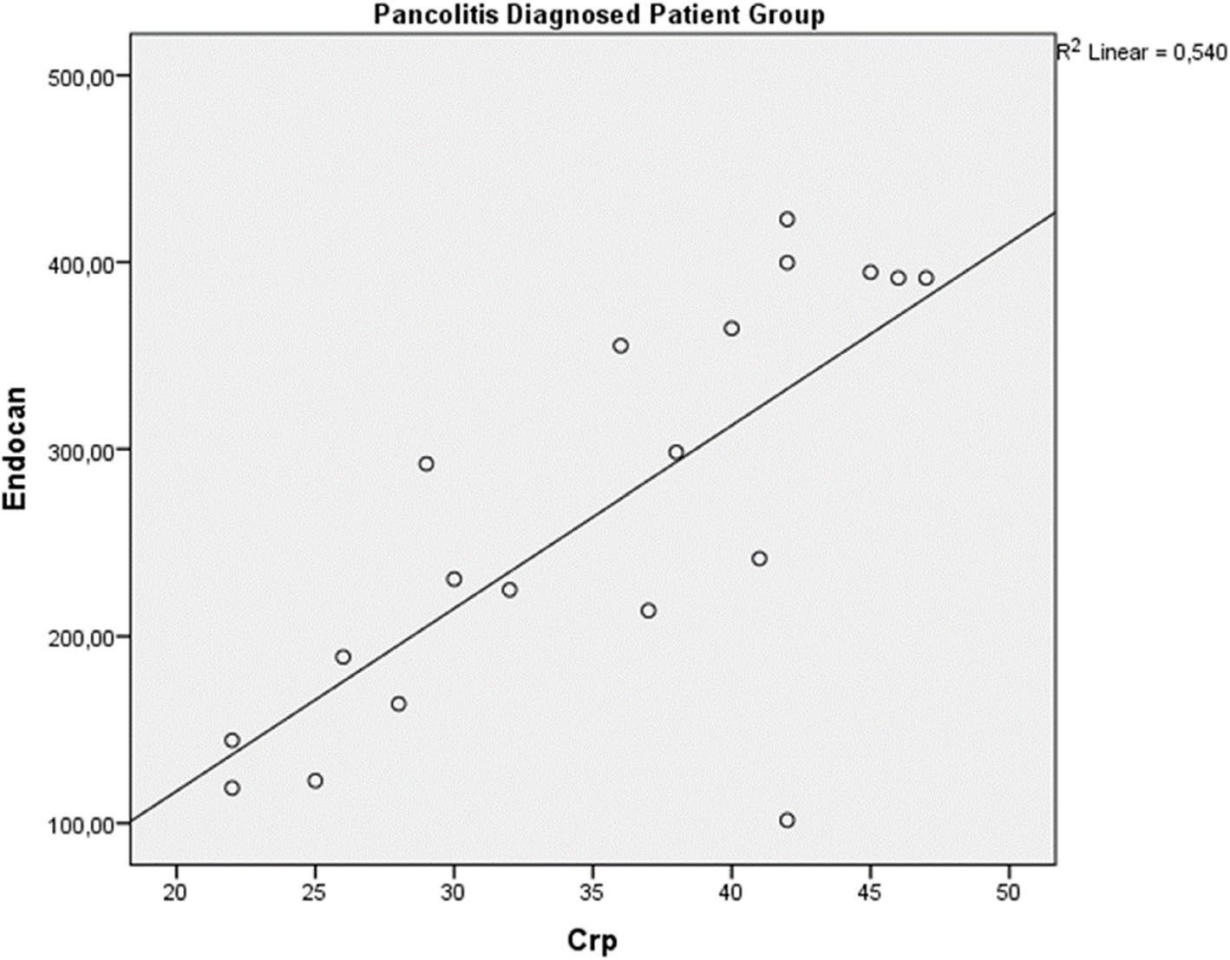
Correlation analysis between Endocan and CRP in a group of patients with pancolitis

There was a significant correlation between endocan and CRP in the patient group (*r*^2^ = 0.78), and also there was a significant correlation between endocan and CRP in patients with extensive colitis (*r*^2^ = 0.54) (Figs. 1 and 2). According to Spearman’s Ranking Correlation Coefficient analysis in the patient group; There was a positive and significant correlation between Endocan and CRP (r_spearman_ = 0.888, *p* < 0.01). There was no correlation between MES and Endocan and CRP. (respectively, r_spearman_ = 0.088, *p* = 0.616 and r_spearman_ = 0.194, *p* = 0.263).

**Fig. 3:**
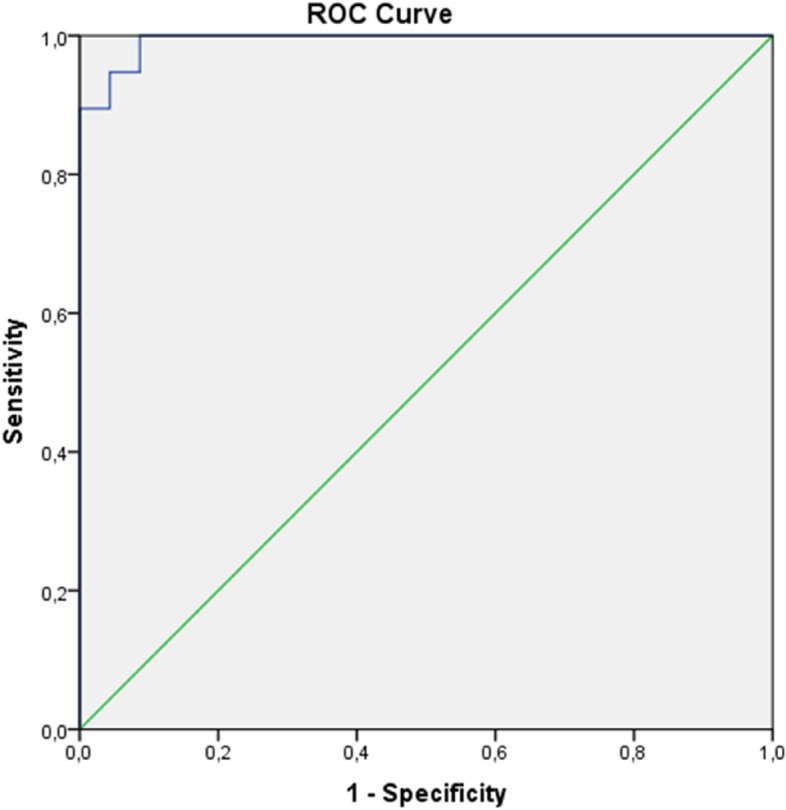
ROC analysis for the endocan cut-off value

According to the ROC analysis, the cut-off value of the endocan level was found to be 95.2 pg/ml using Youden’s index (Fig. 3). The endocan level can be used as a parameter in the detection of diffuse colitis (*p* < 0.001) (Area: 99.3%).

## Discussion

Inflammatory bowel disease (IBD) is characterized by chronic inflammation of the gastrointestinal tract and is associated with risks of complications such as toxic megacolon, surgeries that affect the quality of life, and frequent hospitalizations. It has been stated in studies that more research is needed to measure the extent of inflammatory bowel disease at the appropriate time and to evaluate its effectiveness with one-on-one trials. With timely assessments and treatments, these quality improvement efforts will ultimately improve the quality of care and contribute to the reduction of preventable hospitalizations and healthcare costs from delayed inflammatory bowel disease treatment. Our study is one of the preliminary studies which is conducted to detect the extent of the disease in patients with ulcerative colitis by noninvasive methods with a serum marker such as endocan [1, 3, 8].

Studies conducted in many centers in both infection and inflammatory conditions have revealed that endocan is a strong marker. Wu et al. determined the acid endocan level in patients with decompensated cirrhosis with spontaneous bacterial peritonitis compared to patients with ascites without peritonitis, likewise in Zuwala-Jagiello et al. as a mediator of the inflammatory state associated with a bacterial infection in liver cirrhosis, Omma et al. showed that endocan was significantly higher in colchicine-resistant Familial Mediterranean Fever patients compared to colchicine responders [9–12].

In a study by Voiosu et al. [13] in 33 patients with inflammatory bowel disease, endocan levels were significantly higher in the IBD group when compared with both the healthy control group and cancer patients. The coexistence of both Crohn’s patients and ulcerative colitis patients in the study indicates that our study may be more valuable. Considering that the endocan level is high in patients with IBD, the fact that this elevation mostly indicates patients with pancolitis adds value to our study. Considering that the endocan level is high in patients with IBD, the fact that this elevation mostly indicates patients with pancolitis adds value to our study.

As the extent and severity of ulcerative colitis increase, inflammatory markers in serum and stool increase. In important diseases such as acute pancreatitis with similar inflammation, studies have shown that the level of endocan increases as the severity of the disease increases [14]. In particular, the study of Çakır et al. [15] in infants with necrotizing enterocolitis showed that the endocan increased significantly in showing the severity of the disease, opening a new window. The most important finding in the study of Sakuraba et al. [16] in 133 patients with ulcerative colitis was that the extent of the disease may affect the correlation between fecal biomarker values. Especially in patients with proctitis, the low correlation between stool biomarkers and endoscopic findings, just as in our study, shows that both endocan and fecal calprotectin are more valuable in cases of extensive disease.

In the study of Kyle et al. [17], the fecal marker was used instead of the serum marker to determine the extent of the disease. They found that fecal calprotectin was significantly elevated in a concentration-dependent manner, which correlated with the number of sites of active inflammation reported in colon biopsies from inflammatory bowel patients. As in our study, a comparison was made with CRP. Although CRP and leukocyte measurements showed an upward trend in line with inflammation reported from biopsy, results were highly variable, underscoring the poor reliability of these biomarkers in demonstrating IBD inflammation. In addition, CRP may be elevated due to many factors unrelated to IBD, such as infection, rheumatoid arthritis, and autoimmune diseases. Current blood and serological laboratory tests such as total leukocyte count, C-reactive protein (CRP), and erythrocyte sedimentation rate (ESR), provide indirect, objective but non-specific markers for UC. Few studies have shown relatively poor sensitivity and specificity for the use of these biomarkers in diagnosing IBD and monitoring treatment [18].

Colonoscopy is extremely valuable both in diagnosing patients with inflammatory bowel diseases such as ulcerative colitis (UC) and in evaluating the effectiveness of treatments. Colonoscopy is the gold standard to characterize ulcerative colitis and is used to directly evaluate ongoing mucosal inflammation. Sometimes a colonoscopy may be required to determine disease activity. This procedure is expensive, invasive, and associated with patient preparation, waiting times, and discomfort while carrying a small but significant risk of complications. Therefore, a serum marker such as endocan or a stool marker such as fecal calprotectin can be used both to monitor patients and to decide on treatment for the disease. In addition, the lack of a cut-off value of fecal calprotectin indicating the extent of ulcerative colitis may increase the importance of our study [1, 6, 19].

The missing aspect of our study is that endocan cannot be evaluated in exact comparison with fecal calprotectin because of the cost.

In conclusion, serum endocan level can be useful in determining the extent of ulcerative colitis and planning treatment. It would be appropriate to conduct a large-scale study with a larger number of patients.

## Data Availability

All data generated or analyzed during this study are included in this published article.
